# NanoBiT screening identifies the azaoxafluorene VT11 as potent tau interaction inhibitor

**DOI:** 10.1038/s41598-026-65368-w

**Published:** 2026-08-04

**Authors:** Niya Ma, Jens Stieler, Isabel Hilbrich, Tim Vogel, Andreas Hilgeroth, Detlef Briel, Michael Schaefer, Max Holzer, Moritz Metelmann

**Affiliations:** 1 Paul Flechsig Institute, Centre of Neuropathology and Brain Research, University of Leipzig, Leipzig, Germany; 2 Institute of Pharmacy, Martin Luther University Halle-Wittenberg, Halle, Germany; 3 Faculty of Life Sciences, University of Leipzig, Leipzig, Germany; 4 Rudolf-Boehm-Institute of Pharmacology and Toxicology, University of Leipzig, Leipzig, Germany; 5 Department of Neurology, Medical Faculty, University of Leipzig, Liebigstrasse 20, 04103 Leipzig, Germany; 6 Present Address: Fourth Affiliated Hospital of Guangxi Medical University, Liuzhou, Guangxi China

**Keywords:** Tau-tau interaction, NanoBiT assay, RT-QuIC, Azaoxafluorene VT11, Biochemistry, Drug discovery, Neuroscience

## Abstract

Tauopathies are neurodegenerative disorders characterized by accumulating misfolded, insoluble tau protein aggregates in neurons or glial cells. In this study, we screened the *Spectrum Collection* and other compound libraries for inhibitors of tau self-interaction using a structural complementation reporter system (*NanoLuciferase Binary Technology*). Resulting candidates were tested in dose-response assays and evaluated for cell toxicity and microtubule destabilization. Further, a seed-induced tau interaction biosensor assay and a cell-free tau Real-Time Quaking-Induced Conversion assay have been established to study their effects on the kinetics of tau interaction and aggregation, respectively. The substances ritanserin, 3-methoxycatechol, gambogic amide, azaoxafluorenes VT11, and NS 185 and thieno[2,3-d][1.3]oxazine B6/55 showed a concentration-dependent tau self-interaction inhibition without relevant cell toxicity or microtubule destabilization. Ritanserin, VT11, NS 185 and B6/55 blocked tau interaction in the seed-induced tau interaction biosensor assay. Finally, the cell-free tau RT-QuIC assay displayed highest inhibitory potential for VT11. Thus, the azaoxafluorene VT11 seems to be a promising candidate for further investigations as tau interaction inhibitor to address a pivotal pathological process in Alzheimer’s disease and other tauopathies.

**Supplementary Information:**

The online version contains supplementary material available at https://doi.org/10.1038/s41598-026-65368-w.

## Introduction

Tauopathies are a class of neurodegenerative disorders distinguished by the accumulation of misfolded, filamentous aggregates with cross-β-sheet structure of a protein known as microtubule-associated protein tau (MAPT) in neurons and/or glial cells^1,2^. Tauopathies result in diverse manifestations including cognitive/behavioral disorders, movement disorders, language disorders, and non-specific amnestic symptoms in older individuals^3^. Despite the high prevalence of tauopathies and the considerable burden they impose on patients, caregivers, and healthcare systems, current treatments are merely capable of alleviating symptoms and do not halt disease progression^4^.

On the molecular level, human tau protein, encoded by the *MAPT* gene, is a natively unfolded protein essential for regulating microtubule stability and bundling, axonal integrity, and signaling pathways^5^. In pathological situations, accompanied by aberrant posttranslational modifications, tau undergoes misfolding into β-sheet-rich aggregates in the cytosol triggering the fibrillogenesis cascade^6^. The aggregation process starts from monomers, forming dimers to tau oligomers, which then via protofilaments assemble into paired helical or straight filaments and ultimately lead to intracellular neurofibrillary tangles (NFTs) in Alzheimer’s disease. It is a multistep process with dimerization as the rate-limiting step^7,8^. To prevent the formation of toxic tau oligomers it is feasible to inhibit direct tau-tau interaction.

Thus, the project aimed to identify tau interaction inhibitors from different compound libraries (e.g. *Spectrum Collection* library), which can diminish tau self-interaction, using the NanoBiT tau interaction assay. Selected inhibitors were scrutinized hierarchically for low cellular toxicity, low microtubule destabilization, efficacy for the 3- (1N3R) and 4-repeat (1N4R) tau isoforms, and, in addition, predicted BBB permeability to eliminate unfavorable inhibitors.

Selected candidates were further evaluated in two independent tau interaction assays for their inhibitory capability: (a) a cellular seed-induced tau interaction assay and (b) a cell-free tau seed amplification assay.

## Methods

### Ethical statement

All methods were performed in accordance with the relevant guidelines and regulations. The animal handling was approved by local authorities (T13/16; IRB: Landesdirektion Leipzig), and all animal handling procedures were carried out according to Directive 2010/63/EU of the European Parliament and of the Council on the protection of animals used for scientific purposes.

## NanoLuc binary technology

### Construction of plasmids

The NanoBiT luciferase complementation system (Promega, Madison, WI, USA) was used for protein interaction analysis. The system includes split NanoLuc luciferase, expression vectors (pFN33/35, pFC34/36), positive control constructs (SmBiT-PRKACA and LgBiT-PRKAR2A), and a negative control construct (SmBiT-HaloTag). Constitutive NanoLuc was expressed by the vector pNL1.1.TK[Nluc/TK]. Plasmids were constructed as previously described^9,10^.

### Cell culture and transfection

The well-established human hepatocellular carcinoma cell line HuH-7^9^(Sigma, Ordering No. #01042712-1VL, safety standard S1) was maintained in Dulbecco’s modified Eagle’s medium (DMEM/F-12) supplemented with 10% fetal bovine serum.

Transfection of the indicated constructs in HuH-7 cells was performed in 6-well plates using Lipofectamine 2000 (Thermo Fisher Scientific) according to the manufacturer’s instructions. Briefly, 2.5 µg plasmid DNA (1.25 µg pFN33-tau and 1.25 µg pFC36-tau) was diluted in 250 µl Opti-MEM, while 6.25 µl Lipofectamine 2000 was diluted in a separate 250 µl Opti-MEM. The mixtures were incubated for 20 min at room temperature and then combined before being added to the cells.

Twenty-four hours after transfection, cells were transferred to 384-well plates for the luciferase assay. In cases of slower cell growth, cells were incubated for an additional day before transfer.

### NanoBiT assay

The NanoBiT assay for quantification of tau protein interaction was performed in living HuH-7 cells in 384-well plates. The NanoLuc substrate furimazine (stock solution #N1130, diluted 1:800 in Opti-MEM) was added at 5 µl per well, resulting in a final volume of 35 µl. In our experiments, furimazine provided a stable measurement window of 15–60 min after addition, as previously reported^10,9^. Luminescence was measured at 37 °C using a Mithras LB 940 multimode microplate reader (Berthold Technologies, Bad Wildbad, Germany).

### Screening for tau self-interaction regulators from the Spectrum Collection and rational-designed inhibitors

Compounds from the *Spectrum Collection* were screened using a single-well measurement as done previously with NanoBiT assay^10^. They were dispensed in 384-well plates at a concentration of 10 mM in 100% DMSO and stored at − 80 °C. Mother plates were thawed once to generate four sets of daughter plates (concentration of compounds 100 µM maintained in 100% DMSO), one of which was used for screening. Azaoxafluorene derivatives with protein kinase inhibitory activity were obtained from the pharmaceutical chemistry group of Dr. Andreas Hilgeroth (Martin Luther University Halle-Wittenberg) and thieno[2,3-d]pyrimidine derivatives as glutamate receptor antagonists from Dr. Detlef Briel (Leipzig University). These compounds were stored at 12 mM or 20 mM in 100% DMSO or H_2_O at − 80 °C.

HuH-7 cells were transfected with a 1:1 mixture of pFN33-1N3R_tau and pFC36-1N3R_tau (or pFN33-1N4R_tau and pFC36-1N4R_tau) plasmids for the tau self-interaction assay, along with constitutive NanoLuc pNL1.1.TK[Nluc/TK] as control for the compound interference assay, in 6-well plates. 24 h after transfection, cells were seeded into 384-well plates.

To obtain baseline luminescence readings before treatment, 5 µl of diluted furimazine (1:800 in Opti-MEM, stock #N1130) was added to each well containing 30 µl cell medium, followed by luminescence measurement 0.5 s/well. Subsequently, compounds from the *Spectrum Collection* (final concentration 10 µM; final DMSO concentration 0.1%) were added and incubated for 60 min.

After incubation, 5 µl of diluted furimazine was added again, and luminescence was measured a second time. Finally, the WST-8 assay was performed by adding of 5 µl WST-8 reagent and a 30 min incubation step at 37 °C before reading absorbance at 450 nm to assess compound-induced cytotoxicity.

### Concentration-response verification experiments with selected candidate inhibitors

To validate potential tau aggregation inhibitors, ritanserin, 3-methoxycatechol, gambogic acid amide, VT11, NS 185, and the GluR6 antagonist B6/55 were selected for concentration–response analysis.

HuH-7 cells transfected with pFN33-1N4R_tau and pFC36-1N4R_tau were seeded into 384-well plates, as described for the screening assay, and treated with the indicated compounds.

Compounds from the *Spectrum Collection* were tested at final concentrations of 3.125, 6.25, 12.5, 25, and 50 µM (or 11.11, 16.66, 25, 37.5, and 56.25 µM), whereas rationally designed inhibitors were tested at 1.563, 3.125, 6.25, and 12.5 µM, based on the initial screening results. Cell viability was assessed using the WST-8 assay as described below. To exclude false-positive effects due to altered protein expression, TK-promoter activity, or direct interference with luciferase activity, control vectors encoding established protein interactors S100A/S100B and HBA2/HBB (SmBiT-S100A/HBA2 and LgBiT-S100B/HBB)^11^ were measured in parallel.

### WST-8 cell viability assay

Cell viability was assessed using the WST-8 assay as previously described^10^. WST-8 (2-(2-methoxy-4-nitrophenyl)-3-(4-nitrophenyl)-5-(2,4-disulfophenyl)-2 H-tetrazolium, monosodium salt) was prepared at 5 mM in 150 mM NaCl and supplemented with 200 µM 1-methoxy-PMS (1-methoxy-5-methylphenazinium methyl sulfate).

A total of 10 µl WST-8/1-mPMS mixture was added to 100 µl medium in 96-well plates or 5 µl to 50 µl medium in 384-well plates. After incubation for 30–60 min, depending on cell density, absorbance was measured at 450 nm using a Mithras LB 940 multimode microplate reader (Berthold Technologies, Bad Wildbad, Germany).

### Cell-based model for testing microtubule stability

The assay was adapted from a previously published protocol^12^. HuH-7 cells (1 × 10^4 per well) were seeded in 96-well plates in 100 µl complete medium and incubated at 37 °C with 5% CO₂.

24 h after seeding, nocodazole was added at final concentrations of 1, 2, or 5 µM for 30 min. Cells were then permeabilized for 10 min using OPT buffer (80 mM PIPES, 1 mM EGTA, 1 mM MgCl₂, 0.5% Triton X-100, 10% glycerol, pH 6.8) pre-warmed to 37 °C.

After permeabilization, cells were fixed immediately with prewarmed 4% formaldehyde in PBS (pH 7.2). Cells were washed three times with PBS containing 0.1% Tween-20 and then blocked with 0.1% Tween-20 and 0.5% BSA in PBS for 30 min.

The primary antibody (DM1A anti-α-tubulin, Sigma T6199) was applied for 45 min at the indicated dilution. After washing, HRP-conjugated anti-mouse secondary antibody (Cell Signaling #7076, 1:5000) was added for 45 min.

Following washing, 50 µl ECL substrate (Pierce #32106) was added to each well, and luminescence was measured after 5 min.

For assessment of microtubule stability modulators, cells were treated 24 h after seeding with candidate inhibitors (10 µM, 4 replicates) for 2–24 h. Paclitaxel (0.5 µM) and nocodazole (1 µM) were used as stabilizing and destabilizing controls, respectively.

Subsequent processing steps were performed as described above. DM1A anti-α-tubulin antibody (1:5000) was used as the primary antibody for this assay.

### Cell-free tau RT-QuIC assay

The RT-QuIC assay was performed according to previously described protocols^12^ with minor modifications. The K12 tau fragment (residues 244–275 and 306–400 of numbering according to human 2N4R tau, harboring a C322S mutation; designated K12CF) was cloned into the pET28 vector, resulting in an N-terminal His-tagged construct (K12CFh). In addition, the cysteine-free K18 fragment (K18CF) carrying serine at residues cys 291 and cys 322 was used (Suppl. Figure 1).

Recombinant K12CF and K18CF proteins were purified and used in aggregation buffer containing 50 mM HEPES, 250 mM sodium citrate (pH 7.4), protease inhibitors (cOmplete, Roche), 50 mM dextran sulfate, and 10 µM thioflavin T (ThT). The mixture was ultracentrifuged at 100,000 × g for 30 min at 4 °C to remove potential preformed aggregates.

A total of 95 µl of the reaction mixture was added to each well of a 96-well black µclear plate (Greiner bio-one #655090). Brain homogenates (10% in PBS) from transgenic P301L mice were thawed, diluted in ice-cold PBS, and added to the wells (5 µl per well; corresponding to approximately 0.1 µL of the original brain extract) in quadruplicate or octuplicate. Brain homogenates from tau knock-out mice were used as negative controls.

Three ceramic beads (0.8 mm diameter) were added to each well to enhance quaking-induced amplification. Plates were sealed with adhesive film and incubated in a FLUOstar Omega microplate reader (BMG Labtech). Fluorescence kinetics were monitored with bottom optics using 448/482 nm filters, and tau seeding activity was quantified using a four-parameter logistic (4PL) model.

## Seed-induced tau aggregation biosensors

### Preparation of tau seeds from mouse brain lysates

Brain-derived amyloidogenic tau seeds were obtained from JNPL3 transgenic (P301L) mice^13–15^(Taconic Biosciences, #2508 “STOCKTg(Prnp-MAPT*P301L)JNPL3Hlmc). Five two-month-old male mice were killed by cervical dislocation. The brains were rapidly removed, dissected brainstem flash-frozen in liquid nitrogen.

Frozen brainstem tissue was homogenized in PBS supplemented with cOmplete protease inhibitors (Roche) and EDTA using precooled Precellys homogenizer tubes (CK14 kit). Homogenates were centrifuged at 2,000 × g for 15 min at 4 °C, and the supernatant was collected. The pellet was resuspended again in PBS to the original volume and centrifuged again under the same conditions. Supernatants from both centrifugation steps were combined and further centrifuged at 21,000 × g for 15 min at 4 °C.

The final supernatant was collected, aliquoted, and stored at − 80 °C. Protein concentration (4.6 mg/ml) was determined using a Pierce BCA Protein Assay Kit (Thermo Fisher Scientific).

### Establishing seed-induced tau aggregation biosensors

LgBiT of NanoLuc was fused to the N-terminus of tau, and SmBiT to the C-terminus. Full-length wild-type tau (WT-tau), corresponding to the 1N3R and 1N4R isoforms, as well as the tau fragment dGAE, were used (Suppl. Figure 6 A). Transfected HuH-7 cells expressing NanoBiT tau sensors (LgBiT-1N3R/SmBiT-1N3R, LgBiT-1N4R/SmBiT-1N4R, and LgBiT-dGAE/SmBiT-dGAE) were incubated in 384-well plates.

For tau seeding experiments, brain lysate from tau P301L mice (up to 1.5 µg total protein per well) were mixed with Lipofectamine 2000 (0.125 µl per well) in 5 µl Opti-MEM medium and incubated for 20 min prior to addition to the cells, as previously described^16^.

The Nano-Glo luciferase substrate furimazine was added 24 h after seed transduction at a final dilution of 1:800 in PBS, and luminescence was measured using a Mithras LB 940 reader.

For analysis, luminescence readings at 5 min after substrate addition were used unless otherwise specified.

### Seed-induced tau aggregation biosensor for identification of aggregation inhibitors

HuH-7 cells were transfected with a 1:1 mixture of LgBiT-dGAE and SmBiT-dGAE in 6-well plates. Twenty-four hours after transfection, cells were reseeded into 384-well plates.

Tau seeds derived from tau P301L mice (up to 1.5 µg total protein per well) were pre-incubated with Lipofectamine 2000 for 20 min and then added to cells expressing tau constructs. Candidate inhibitors (10 µM) were added subsequently and cells were incubated for 24 h. DMSO (0.1%) was used as a vehicle control.

The NanoLuc substrate furimazine (stock solution #N1130, diluted 1:800 in Opti-MEM) was added to achieve a final volume of 35 µl per well, and luminescence was measured.

Subsequently, 5 µl of WST-8 reagent was added and incubated for 30 min, and absorbance was measured at 450 nm using a Mithras LB 940 reader.

### Statistical analysis

Statistical analysis was performed using GraphPad Prism (version 10). Data were presented as mean ± standard deviation (SD). Luminescence data were analyzed as raw values (arbitrary units) or normalized to the baseline signal.

Normality of data distribution was assessed using the Shapiro–Wilk test. For comparisons between two groups, the Mann–Whitney U test was applied when data were not normally distributed, unless otherwise specified. For comparisons among multiple groups, one-way ANOVA followed by Dunnett’s multiple comparison test was used. For RT-QuIC data analysis and curve fitting, SigmaPlot (version 13.0) was used. A p value < 0.05 was considered statistically significant. Statistical significance levels were defined as follows: *p < 0.05, **p < 0.01, ***p < 0.001, and ****p < 0.0001.

## Results

### Screening for inhibitors of tau self-interaction

Screening has been based on the sensitive split-nanoluc technology, where even transient protein-protein interaction can be detected^17^. Configuration of NanoLuc tau fusion proteins has been tested and resulted in fusion of the large bit (pFN33) and the small bit (pFC36) to N-terminus and C-terminus, respectively, of 1N3R and 1N4R tau isoforms with the highest luciferase activity (*p* < 0.005, Suppl. Figure 2).

The *Spectrum Collection* was used to identify potential inhibitors of tau self-interaction, because it brings together FDA-approved compounds, compounds that have progressed to Phase III clinical trials, natural compounds and frequently used cell biological tools. Besides, rational-designed protein kinase inhibitors from the azaoxafluorene scaffold^18,19^ as well as thieno[2,3-d]pyrimidine compounds^20^ have also been tested. Recently, thiophene-containing heterocycles have been shown to bind to tau aggregates and inhibit tau aggregation^21,22^.

**Fig. 1:**
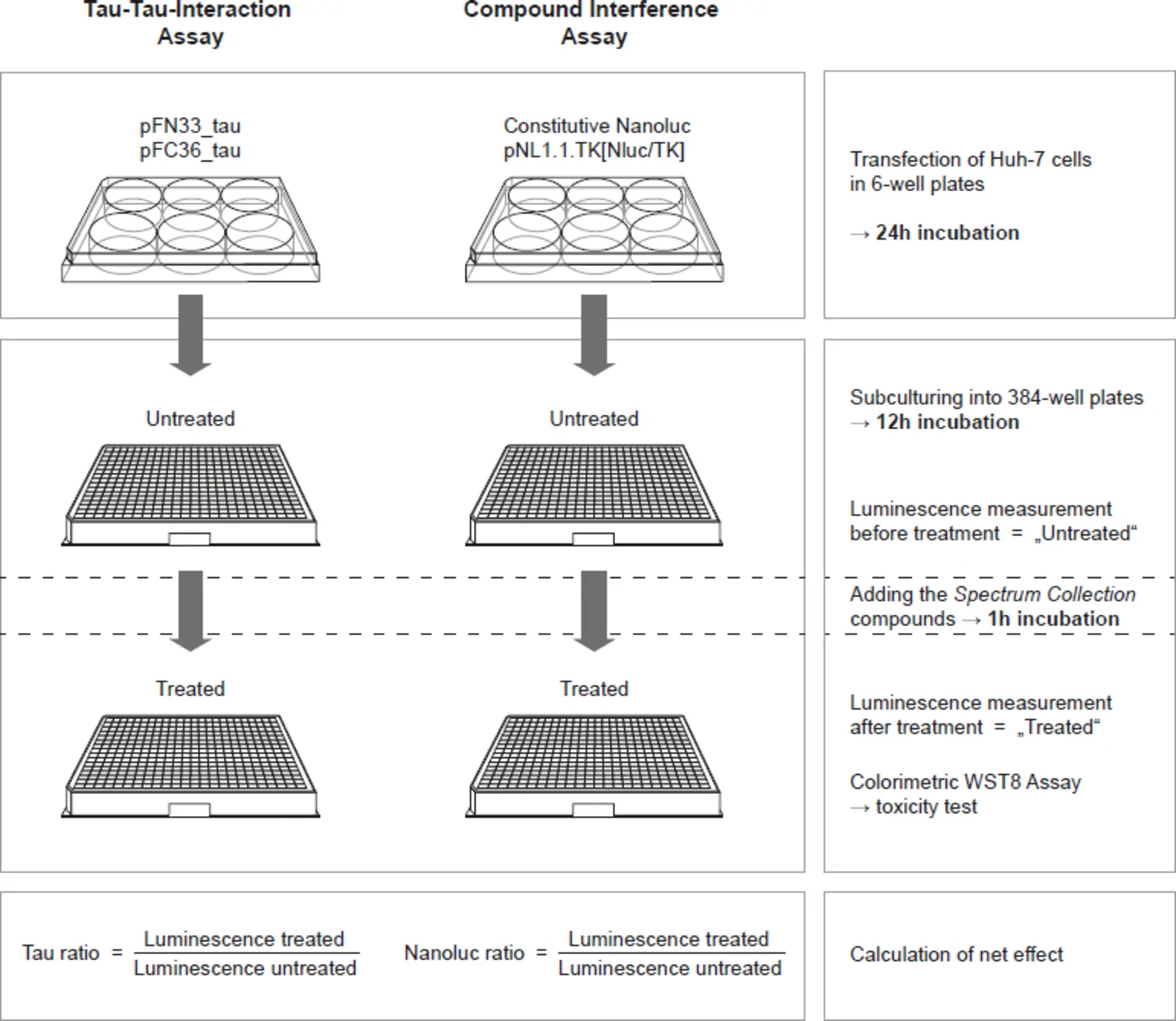
Flowchart of NanoBiT tau interaction measurement with the *Spectrum Collection* and the rationally designed inhibitors screen. The HuH-7 cells were seeded on a 6-well plate, 24 h later transfected with the split NanoLuc plasmids LgBiT and SmBiT-fusion tau protein using the Lipofectamine 2000 transfection reagent. The HuH-7 cells were then subcultured into a 384-well plate after 24 h. Furimazine was added 12 h later and a baseline luminescence reading of untreated cells was started. This is needed to compensate for well to well variation in cell number. After cell medium removal, cells were incubated with screening compound containing medium for 1 h before a second luminescence reading again with furimazine addition. The change of luminescence signal between baseline and treated cells was used to calculate the tau ratio. To detect off-target effects of compounds, such as interference with luciferase reaction, light emission, protein homeostasis, and TK promoter activity, we applied the same compounds onto HuH-7 cells transfected with a construct encoding constitutive, full-length NanoLuciferase (Compound Interference Assay) yielding the nanoluc ratio, which should ideally close to 1.0, when no interference is present.

The flowchart of NanoBiT tau interaction screening is shown in Fig. 1. Compounds causing a reduction of tau self-interaction by at least 30% were considered inhibitors, whereas compounds increasing the interaction by 20% or more have been considered as potential stimulators according to previous publications^10,9^. In the primary screen, 42 hits were identified as potential inhibitors (Suppl. Table 1).

Inhibitors were validated in a secondary screen, applying final concentrations of 3.125 µM, 12.5 µM, and 50 µM for NanoBiT tau interaction assay and cell viability. The tau self-interaction was tested before and 1 h after treatment whereas toxicity was estimated with the WST-8 cell viability assay. Of the primary hits, six compounds met the rigorous verification criteria with inhibition of both 3R and 4R tau self-interaction at 10 µM concentration: ritanserin, gambogic amide, 3-methoxycatechol, VT11, NS 185 and B6/55 (Table 1). Ritanserin (24% and 29% for 1N3R and 1N4R tau protein isoforms respectively), gambogic amide (52% and 59% for 1N3R and 1N4R tau protein isoforms respectively), 3-methoxycatechol (70% for 1N3R tau protein isoforms), VT11 (48% and 69% for 1N3R and 1N4R tau protein isoforms respectively), NS 185 (41% and 54% for 1N3R and 1N4R tau protein isoforms respectively), and B6/55 (47% and 17% for 1N3R and 1N4R tau protein isoforms respectively) showed sufficient tau interaction inhibition (Table 1). To explore BBB permeability of the potential inhibitors, small-molecule pharmacokinetics prediction via pkCSM was chosen in our study. Except gambogic amide (logBB = -0.882) and 3-methoxycatechol (logBB = -0.314) all other candidates were predicted to sufficiently permeate through the BBB.

**Fig. 2:**
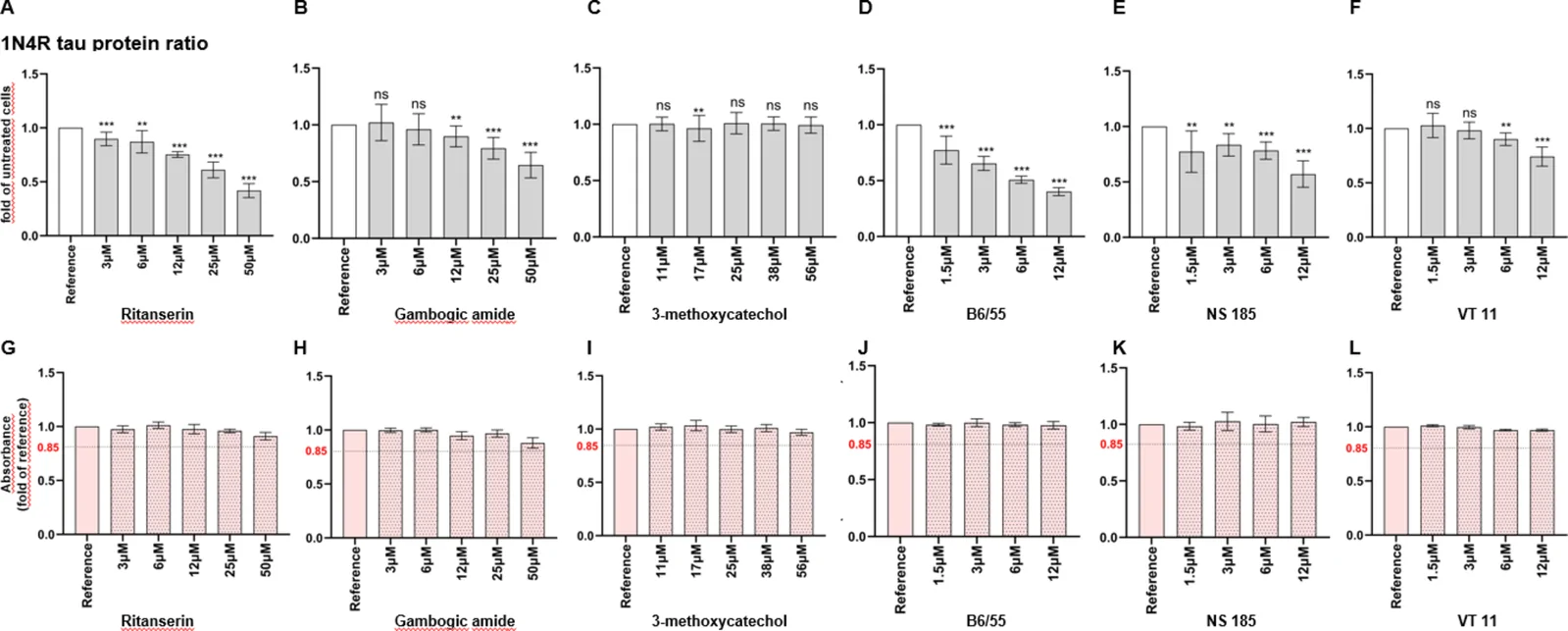
Dose-response curve of 4R tau self-interaction after treatment with ritanserin, gambogic amide, 3-methoxycatechol, B6/55, NS 185, and VT11 for 1 h. (A–F) NanoBiT tau interaction assay showing the tau self-interaction after treatment with ritanserin (**A**), gambogic amide (**B**), 3-methoxycatechol (**C**), B6/55 (**D**), NS 185 (**E**), and VT11 (**F**) at the indicated concentrations. (G–L) Cell viability assessed by WST-8 assay following treatment with ritanserin (**G**), gambogic amide (**H**), 3-methoxycatechol (**I**), B6/55 (**J**), NS 185 (**K**), and VT11 (**L**). Data are expressed as percentage of untreated control (mean ± SD). Statistical significance was determined using the Mann–Whitney U test (ns, *p* > 0.05; **p < 0.01; ***p < 0.001; *n* = 4–8 technical replicates).

Ritanserin showed a concentration-dependent reduction of tau interaction by 10% (3.125 µM), 13% (6.25 µM), 25% (12.5 µM), 39% (25 µM), and 58% (50 µM) and exerted little cellular toxicity (all cell viability > 0.85) (Fig. 2A, G). Gambogic amide treatment resulted in a concentration-dependent reduction of tau interaction by 4% (6.25 µM), 10% (12.5 µM), 21% (25 µM), and 35% (50 µM) (Fig. 2B, H). 3-methoxycatechol did not lead to a significant reduction of tau interaction at concentrations of 11.1 µM, 25 µM, 37.5 µM, and 56.25 µM, except in concentration of 16.66 µM (Fig. 2C, I). However, after incubation for 24 h 3-methyoxycatechol showed a concentration-dependent reduction of tau interaction by 5% (11.1 µM), 10% (16.66 µM), 15% (25 µM), 21% (37.5 µM) and 21% (56.25 µM) (Suppl. Figure 3).

The thieno[2,3-d][1.3]oxazine B6/55 showed significant and concentration-dependent reduction of tau interaction without relevant cellular toxicity: reduction of tau interaction by 23% (1.56 µM), 35% (3.13 µM), 49% (6.25 µM), and 60% (12.5 µM) (Fig. 2D, J). Similarly, the azaoxafluorene NS 185 showed a significant reduction of tau interaction with less stringent concentration dependence: reduction of 23% (1.56µM), 17% (3.125 µM), 22% (6.25 µM), and 43% (12.5 µM) (Fig. 2E, K). The azaoxafluorene VT11 led to concentration-dependent reduction of tau interaction, starting with significant effect at 6.25 µM without relevant cell toxicity: reduction of tau interaction at concentrations of 2% (3.125 µM), 10% (6.25 µM), and 26% (12.5µM) (Fig. 2F, L).

In summary, ritanserin, gambogic amide, B6/55, and VT11 resulted in a significant and concentration-dependent reduction of tau self-interaction without relevant cell toxicity, while effect of NS 185 was less concentration-dependent and 3-methoxycatechol did affect tau self-interaction after 24 h incubation time.

### Effect of tau interaction inhibitors on microtubule stability

The ideal inhibitor should block tau interaction without impairing the physiological role of tau in stabilizing microtubules, ensuring treatment specificity and minimizing cellular damage.

A quantitative HuH-7 cell-based assay was employed that probed for microtubule stability under treatment with depolymerization agent nocodazole and tau interaction inhibitors (Suppl. Figure 4)^12^.

Ritanserin, gambogic amide, 3-methyloxycatechol, VT11, NS 185, B6/55, and the tau-aggregation inhibitor anle138b^23^ were incubated at 10 µM concentration in the cell-based microtubule stability assay for 2 h and 24 h. Nocodazole resulted in a significant microtubule destabilization. None of the tested inhibitors affected the microtubule stability to a great extent after 2 h, while gambogic amide, 3-methoxycatechol, anle 138b and NS 185 resulted in a significant microtubule destabilization after 24 h (Suppl. Figure 5). Ritanserin, B6/55 and VT11 did not modify microtubule stability also after the long incubation period.

### Kinetics of pathological tau aggregation using a cell-free Tau RT-QuIC assay

A real-time quaking-induced conversion (RT-QuIC) platform was used to study the direct effects of tau interaction inhibitors on tau aggregation in a cell-free setup. The recombinant human 3R tau fragment K12CF or human 4R tau fragment K18CF were exposed to tau aggregates (seeds) from P301L mouse brain homogenate in the presence of different concentrations of the tau interaction inhibitors.

**Fig. 3:**
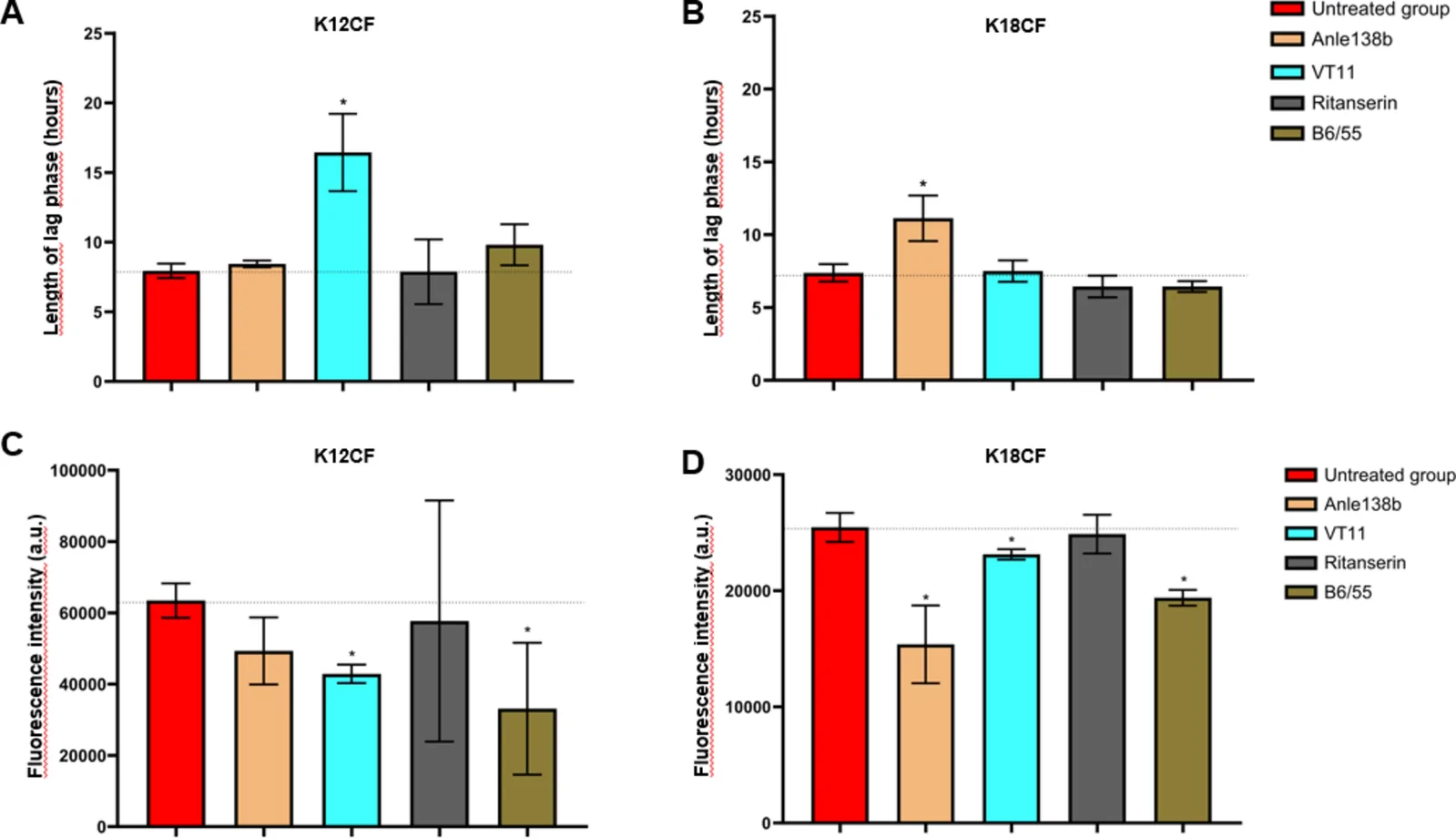
Inhibition of seed-induced tau aggregation in RT-QuIC assay using 3R-tau (K12CF) and 4R-tau (K18CF) by candidate inhibitors. K12CF and K18CF tau RT-QuIC reactions were seeded with tau aggregates from tau P301L mouse brain in the presence of different compounds (anle138b, VT11, B6/55, and ritanserin; 30 µM). (A, B) Length of the lag phase using 3R-Tau K12CF (A) and 4R-tau K18CF (B). (C, D) Fluorescence intensity at plateau in 3R-tau K12CF (C) and 4R-tau K18CF (D). Data are expressed as mean ± SD from two independent experiments. Statistical significance was determined using the Mann–Whitney U test (ns, *p* > 0.05; *p < 0.05; *n* = 4 technical replicates).

Anle138b increased the lag time and decreased the plateau phase amplitude in the 4R tau RT-QuIC assay, but did not show significant effects in the 3R tau assay (Fig. 3). VT11 significantly increased the lag time in the 3R tau assay, and decreased the plateau phase amplitude in both the 3R and 4R tau RT-QuIC tests. B6/55 decreased the plateau phase amplitude in the 3R and 4R tau RT-QuIC assays, but did not show a significant effect on lag times, while ritanserin did not have significant effects on tau aggregation in both RT-QuIC assays (Fig. 3).

In conclusion, VT11 showed the strongest effects on tau aggregation in the cell free assay compared to the other compounds.

### Seed-induced tau aggregation biosensors for identifying tau aggregation inhibitors

A NanoBiT-based biosensor for intracellular tau interaction triggered by extracellular tau seeds was established. The split Nanoluc was fused to the dGAE tau fragment (residues 297–391), a truncated form of tau covering the end of R2, the entire R3, and R4 repeat domains, that has been shown to mimic the core structure of paired helical filaments^24^.

Following expression of the dGAE tau constructs in Huh-7 cells, a robust NanoBiT signal reflecting tau self-interaction was detected upon seeding with tau aggregates derived from brain lysates of P301L mice (all *p* < 0.001; Suppl. Figure 6B).

**Fig. 4:**
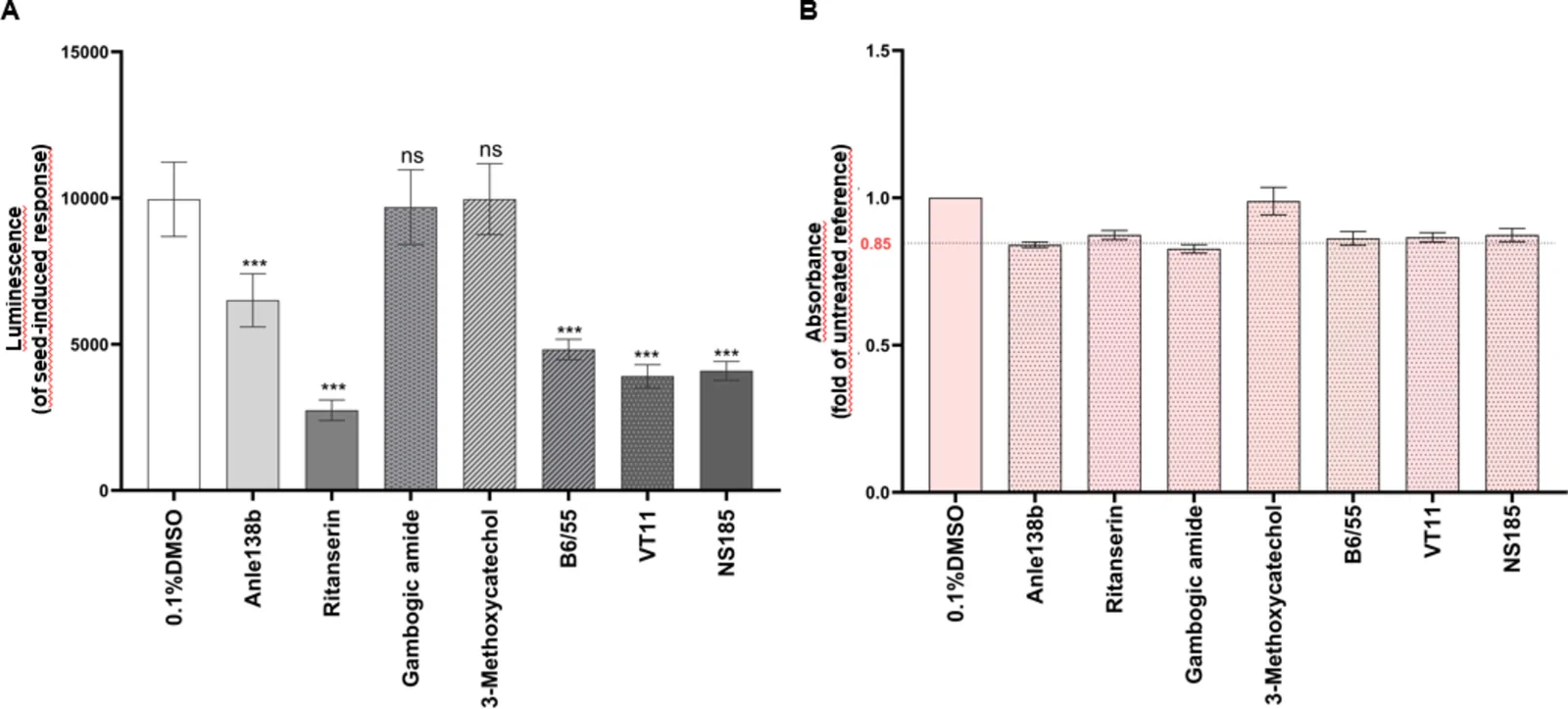
Inhibition of seed-induced tau interaction biosensor for evaluating tau aggregation inhibitors and cell viability. (**A**) Tau P301L mouse brain lysates were applied to the dGAE tau biosensor in the presence of different candidate inhibitors (10 µM). (**B**) Cell viability was assessed using the WST-8 assay 24 h after co-treatment with Tg P301L mouse brain lysates and selected inhibitors (10 µM). Data are expressed as mean ± SD. Statistical significance was determined using the Mann–Whitney U test (ns, *p* > 0.05; *p < 0.05; **p < 0.01; ***p < 0.001; *n* = 8 technical replicates).

To analyze the effect of the selected compounds regarding its interference with intracellular tau aggregation cells transfected with the dGAE tau biosensor were incubated with the compounds for 24 h and simultaneously exposed to seeds from P301L mice brain lysates (Suppl. Figure 7). The compounds anle138b, ritanserin, B6/55, VT11 and NS 185 showed a significant tau aggregation inhibition (Fig. 4A).

Anle138b showed 35% inhibition at 10 µM concentration in dGAE tau biosensors. Ritanserin showed 72% inhibition, whereas the azaoxafluorene VT11 showed 61% inhibition. The azaoxafluorene NS 185 led to 59% inhibition and thieno[2,3-d][1.3]oxazine B6/55 to 52% inhibition, respectively. Gambogic amide and 3-methoxycatechol had no significant effect on the P301L tau seed-induced biosensor read-out. In addition, 24 h after adding candidate compounds no cytotoxicity was observed (Fig. 4B). In conclusion, inhibition of seed-induced tau aggregation has been more pronounced by ritanserin, VT11, B6/55 and NS 185 than by the established tau aggregation inhibitor anle138b.

## Discussion

### Promising candidates of tau interaction inhibition by NanoBiT assay

Dimerization is the rate-limiting step of a multistep process in tau aggregation^7^. Thus, inhibiting direct tau-tau interaction may reduce tau dimerization - disrupting the formation of toxic tau oligomers. In this study, we aimed to identify tau interaction inhibitors employing a sensitive NanoBiT tau interaction assay from different substance libraries.

Six out of 2200 screened compounds showed reproducible inhibition of tau self-interaction for 3R and 4R tau isoforms in concentration-response experiments and no relevant cell toxicity: Ritanserin (serotonin receptor antagonist), 3-methoxycatechol (agonist of G protein-coupled receptor 35), gambogic amide (TrkA agonist), VT11 (GSK3 inhibitor), NS 185 (JNK inhibitor), and B6/55 (GluR6 antagonist).

Beside gambogic amide and 3-methoxycatechol, all tested compounds showed a good predicted BBB permeability as prerequisite to reach their target site. Furthermore, Ritanserin, B6/55 and VT11 did not destabilize microtubules indicating their treatment specificity.

### Seed-induced tau aggregation biosensors for verifying tau interaction inhibitors

The dGAE tau fragment (aa 297–391, 4-repeat tau) employed in the seed-induced tau interaction assay can self-assemble into filaments without the addition of anionic cofactors, and has been found in paired helical filaments in Alzheimer´s disease brains^25^. It has been chosen to investigate seed-induced tau aggregation, because the dGAE fragment (a) represents the protease-resistance of tau fibrils^16^, (b) assembles into Alzheimer fold identical structured fibrils in vitro^24^ and (c) and excludes MT-based effects of tau-tau interaction^26^.

The anti-aggregation compound and positive control anle138b^27^ resulted in 35% inhibition of dGAE biosensor aggregation after seeding with P301L mouse brain lysates – less than about 50% decrease previously described^26^. Inhibition of seed-induced tau aggregation has been even stronger by ritanserin, VT11, B6/55 and NS 185 in the present study. These compounds are reported here for the first time to inhibit seed-induced tau aggregation.

### Cell-free RT-QuIC assay reveals VT11 as a potent tau interaction inhibitor

In recent years, highly sensitive real-time quaking-induced conversion (RT-QuIC) seed amplification assays (SAAs) have been created to detect proteopathic aggregates based on their seeding activities^28,29^. The Tau RT-QuIC assays use the ability of pathological tau aggregates to initiate the misfolding of recombinant tau monomers into ThT-positive tau fibrils. In these reactions, a small amount of self-propagating protein aggregate (known as seed) is mixed with a large amount of recombinant tau monomers (known as the substrate). In this study, we used the tau RT-QuIC assay to test for direct effects of inhibitors on tau protein misfolding and aggregation.

Eventually, only VT11 showed a potential as a direct tau interaction inhibitor by reducing the propagation of amyloidogenic misfolding. It prolonged the lag time before misfolding occurred, reducing the duration of the curve’s plateau, and slowing down growth phases. The effect was much stronger for 3R tau (K12CF tau fragment) than 4R tau (K18CF tau fragment was used). The findings suggest that VT11 may directly interact with tau protein, independently of its kinase inhibitory activity, thereby attenuating pathological misfolding and subsequent β-sheet formation.

Conversely, the previously published tau aggregation inhibitor anle138b shows better inhibitory effect on 4R tau than for 3R tau^26,27,23^.

B6/55 decreased the plateau phase amplitude in the 3R and 4R tau RT-QuIC assays, which implicates less maximal tau aggregation. However, it did not show a significant effect on lag time, which means no prolonged start of detectable aggregate formation.

Ritanserin did not have significant effects on tau aggregation in both 3R and 4R tau RT-QuIC assays. Thus, the inhibitory effect of ritanserin and B6/55 is not mainly based on direct interaction with tau protein. Possibly, their effect is mediated by changes of the cellular metabolism, such as cellular turnover or posttranslational modification; e.g. ritanserin is a potent 5-HT_2A_ receptor antagonist and attenuates protein kinase C phosphorylation^30^. 5-HTR signaling is currently investigated for alternative therapies of tauopathies. Afshar and colleagues demonstrated in a rat model of AD that the use of an 5-HT_1A_ receptor antagonist and 5-HT_2A_ receptor agonist resulted in a significant reduction of oxidative stress and limits loss of hippocampal neurons^31^. Recently, amisulpride, which acts as an inverse agonist on the 5-HT7 receptor has been shown to prevent tau aggregation in vitro and in vivo^32^.

### VT11 as a dual modulator of tau phosphorylation and interaction

VT11, a promising tau interaction inhibitor, is a designed GSK3 inhibitor previously described as compound 3e^9^. VT11 showed reproducible inhibition of tau self-interaction^9^ and now even aggregation inhibitory activity in the cell-free RT-QuIC assay. This suggests that VT11 may interact with monomeric or multimeric tau protein.

Increased activity of GSK-3β can lead to excessive phosphorylation of tau, contributing to neurodegeneration, which makes GSK3 a promising target for therapeutic interventions in AD^33^. Studies in AD amyloid mouse models have shown that blocking GSK-3 using different substances leads to a decrease in the buildup of Aβ, the formation of neuritic plaques, the phosphorylation of tau, and an improvement in cognitive deficits as measured by behavioral tests^34,35^. A recent study indicates that the presence of GSK-3β haploinsufficiency in transgenic mice leads to a decrease in tau hyperphosphorylation, synaptic accumulation, aggregation, and trans-cellular spread in the brain^36^. In recent years, significant efforts have been made to develop small molecules, peptides, and natural compounds targeting GSK-3. Various GSK-3 inhibitors have been found to decrease tau phosphorylation in mice and cells. Although there has been a significant increase in the development of GSK-3 inhibitors in recent years, only few of them have progressed to clinical trials involving human subjects, and none has been approved for therapeutic use. Thus, VT11 may represent a dual-function inhibitor that suppresses tau interaction while simultaneously reducing tau phosphorylation via GSK3 inhibition.

### Limitations

Firstly, the structural complementation reporter system (*NanoLuciferase Binary Technology*) is based on substrate-dependent luminescence in the blue spectrum (~ 460 nm). Certain coloured compounds interfere with luminescence signals, these compounds were detected by our compound interference assay using constitutively active NanoLuc and were excluded from further investigations. Especially, catechol-structure containing compounds which are coloured were therefore omitted from further analyses. Besides, compounds with detergent-like properties were excluded based on the hypothesis that they unspecifically might enhance the luminescence signal.

Single-well measurements during the screening phase may have increased the risk of false positive and false negative hits. Therefore, promising candidates were subsequently validated in concentration–response experiments. However, no further quantitative pharmacological parameters (e.g. IC₅₀ values) have been calculated for concentration-response experiment, which limits comparability of inhibitory potency between the compounds.

Furthermore, the RT-QuIC assay relies on ThT fluorescence to detect amyloidogenic structures. Some compounds may interfere with ThT fluorescence by altering excitation or emission. To minimize this effect, compound-induced fluorescence quenching was assessed using preformed tau fibrils and used to adjust the fluorescence results.

Moreover, tau seeds derived from tau P301L mouse brains were used in this study, which may not fully recapitulate human disease conditions. Human AD-derived tau aggregates may provide a more physiologically relevant seed.

Finally, the molecular mechanisms underlying VT11-mediated tau inhibition remain to be fully elucidated – especially regarding its effect on the different tau isoforms. Future studies should investigate direct interactions between VT11 and tau protein using biophysical approaches, as well as evaluate its in vivo efficacy in relevant animal models.

## Conclusions

In this study, the NanoBiT interaction assay has been used to screen large compound libraries for tau interaction inhibitors. The screen identified several candidate compounds, including ritanserin, 3-methoxycatechol, gambogic amide, the azaoxafluorenes VT11, NS 185, and the GluR5/6 antagonist B6/55. These compounds showed concentration-dependent inhibition of tau interaction without relevant cellular toxicity. Ritanserin, VT11, NS 185 and B6/55 blocked tau aggregation in the dGAE tau aggregate biosensor assay induced by extracellular tau seeds. Finally, a cell-free 3R- or 4R-tau RT-QuIC assay to study the kinetics of tau aggregation showed the strongest inhibitory effect for VT11 by prolonging the lag phase and reducing the amplitude of the sigmoidal aggregation curve. Thus, the azaoxafluorene VT11 may represent a promising candidate for further investigations as potential inhibitor of tau interaction, the underlying pathological process in Alzheimer’s disease and other tauopathies.

**Table 1:**
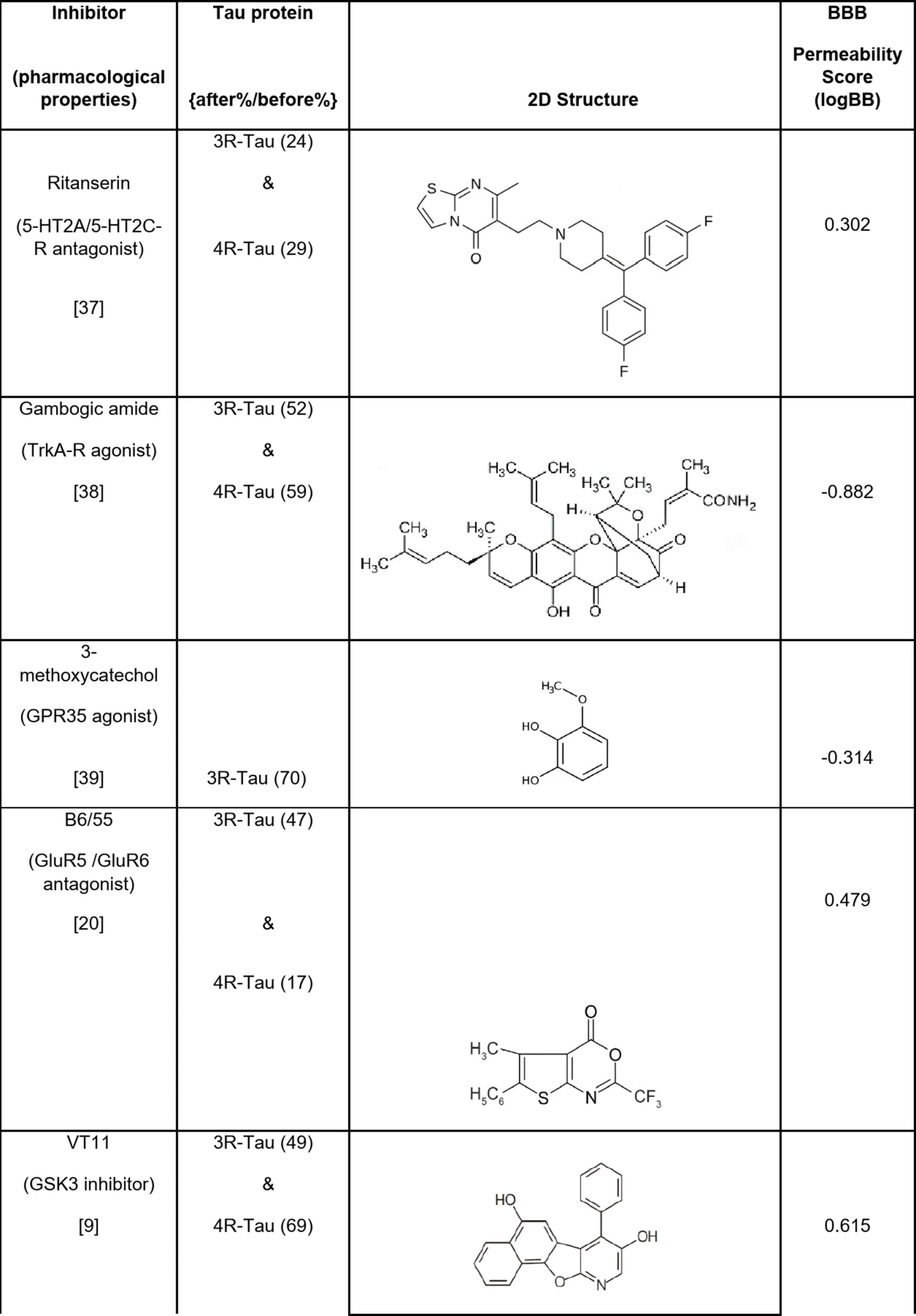

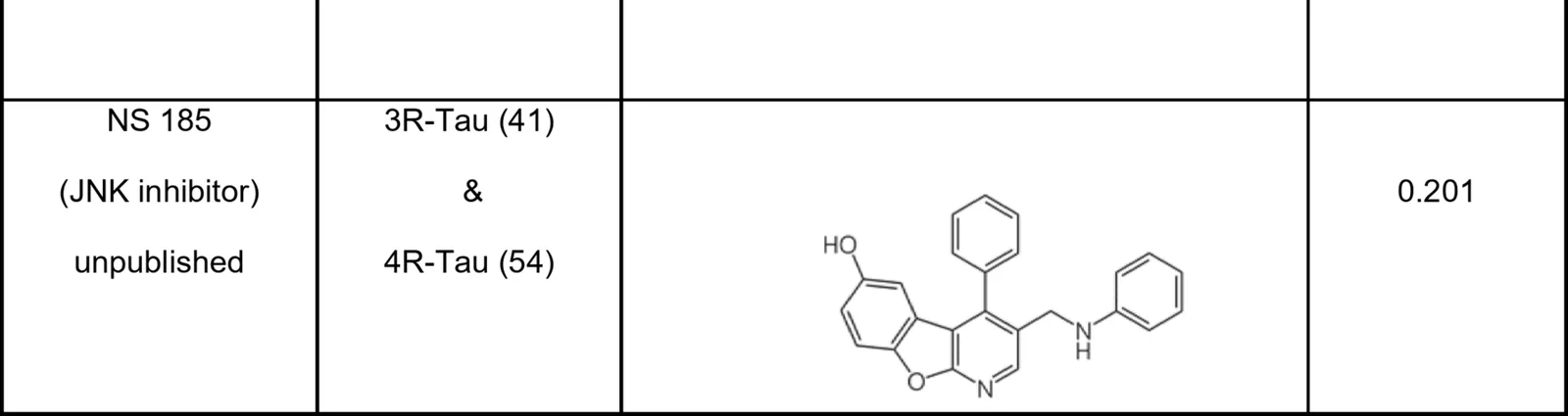
Pharmacological properties, target proteins, chemical structures, and predicted blood–brain barrier (BBB) permeability of the selected compounds. BBB permeability was estimated using the in silico approach pkCSM. Predictions of BBB permeability by pkCSM are provided as logBB values.

## Supplementary Information

Below is the link to the electronic supplementary material.

Supplementary Material 1

## Data Availability

The datasets generated and analysed during the current study are not publicly available but are available from the corresponding author on reasonable request.
