# Supplementary material for: NanoBiT screening identifies the azaoxafluorene VT11 as potent tau interaction inhibitor
Source: Sci Rep. 2026 Aug 4;16:24013. doi: 10.1038/s41598-026-65368-w (PMC13438802; doi:10.1038/s41598-026-65368-w)
Supplement: Supplementary file 1 [file 41598_2026_65368_MOESM1_ESM.docx]

**Supplementary Material**

NanoBiT screening identifies the azaoxafluorene VT11 as potent tau interaction inhibitor

Ma, Niya, Stieler, Jens, Hilbrich, Isabel, Hilgeroth, Andreas, Briel, Detlef, Schaefer, Michael, Holzer, Max, and Metelmann, Moritz

Corresponding author: Moritz Metelmann, MD, PhD, Department of Neurology**,** University of Leipzig, Leipzig, Germany, moritz.metelmann@medizin.uni-leipzig.de

**Supplementary figures**

**Suppl. figure 1**. Molecular structure of recombinant K12CF and K18CF tau. The reference proteins used were htau40 (full-length tau 2N4R, 441 residues, longest CNS isoform) and K12 tau. The K12CF tau comprises the K12 3-repeat tau protein fragment, which includes residues 244-275 and 306-400 from the complete human tau sequence. This fragment is made cysteine-free by an alanine mutation at residue Cys322. The K18CF tau comprises the K18 4-repeat tau fragment, which includes residues 244-372 from the complete human tau sequence. This fragment is made cysteine-free with mutations at residues 291 and 322 cysteine to serine. Modified after [1, 2].

**Suppl. figure 2.** **NanoBiT assay of tau self-interaction.** (A) Interaction of N-terminal (pFN33, LgBiT; pFN35, SmBiT) and C-terminal (pFC34, LgBiT; pFC36, SmBiT) fusion constructs of 1N3R tau in Huh-7 cells. pFN33-S100B/pFC36-S100A interaction served as positive control, and the pair pFN33-Tau/pFN35-Map1 was used as negative control. (B) Interaction of N- and C-terminal fusion constructs of 1N4R tau. pFN33-S100B/pFC36-S100A served as positive control, and pFN33-Tau/pFN35-Map1 as negative control as well. Cells seeded in 384-well plates were analyzed 24 h after transfection. Data are expressed as mean ± SD.

**Suppl. figure 3. Dose-response curve of 4R tau self-interaction after treatment with 3-methoxycatechol for 24 h.** (A) NanoBiT tau interaction assay showing the tau self-interaction after treatment with 3-methoxycatechol at the indicated concentrations. (B) Cell viability assessed by WST-8 assay following treatment with 3-methoxycatechol. Data are expressed as percentage of untreated control (mean ± SD). Statistical significance was determined using the Mann–Whitney U test (ns, p > 0.05; **p < 0.01; ***p < 0.001; n = 4–8 technical replicates).

**Suppl. figure 4. Detection of microtubule destabilization by nocodazole in Huh-7 cells.** Huh-7 cells were seeded in 96-well plates and treated with nocodazole (1, 2, and 5 µM) for 30 min or with 0.1% DMSO as vehicle control. After cell permeabilization and fixation microtubules were detected using DM1A anti-α-tubulin antibody (1:5000). Data are expressed as mean ± SD from two independent experiments (n = 8 technical replicates). Statistical significance was determined using one-way ANOVA (ns, p > 0.05; *p < 0.05; **p < 0.01; ***p < 0.001; ****p < 0.0001).

**Suppl. figure 5. Effect of tau aggregation inhibitors on microtubule stability.** Huh-7 cells were treated for 2 h (A) and 24 h (B) with 0.1% DMSO (vehicle control), paclitaxel (0.5 µM; microtubule stabilizer), nocodazole (1 µM; microtubule destabilizer), or the indicated inhibitors (10 µM), respectively. Microtubules were detected by chemoluminescence using a DM1A anti-α-tubulin antibody (1:5000). Data are expressed as mean ± SD from three to four independent experiments (n = 6 independent wells per condition). Statistical significance was determined using the Mann–Whitney U test (ns, p > 0.05; *p < 0.05).

**Suppl. figure 6. Development of a NanoBiT-based biosensor for seed-induced tau aggregation.** (A) Schematic representation of tau constructs used in the NanoBiT assay, including full-length 1N3R and 1N4R isoforms and the dGAE fragment (297–391) covering the R2–R4 microtubule-binding repeat domain. (B) Tau self-interaction measured by the NanoBiT assay in Huh-7 cells expressing 1N3R, 1N4R, or dGAE constructs 24 h after seeding with tau aggregates derived from brain lysates of P301L tauopathy mice. Data are presented as mean ± SD (n = 8 technical replicates). Statistical significance was determined using the Mann–Whitney U test (ns, p > 0.05; *p < 0.05; **p < 0.01).

**Suppl. figure 7. Workflow for identification of inhibitors of seed-induced tau self-interaction using a NanoBiT-based dGAE biosensor.** Schematic overview of the screening workflow in Huh-7 cells expressing LgBiT-dGAE and SmBiT-dGAE constructs. Cells were incubated with tau seeds derived from tau P301L mouse brain lysates and subsequently added candidate compounds for 24 h, followed by luminescence measurement using NanoLuc substrates.

| **Name** | Tau protein {after%/before%} | **ID** | **Formula** | **Bioactivity** |
| --- | --- | --- | --- | --- |
| **Inhibition** |  |  |  |  |
| ^a^CETRIMONIUM BROMIDE | 3R-Tau(3)&4R-Tau(3) | 1503200 | C19H42BrN | Antiinfectant |
| TACROLIMUS | 4R-Tau(62) | 1503968 | C44H69NO12 | immune suppressant, antifungal |
| ISOXICAM | 4R-Tau(61) | 1503242 | C14H13N3O5S | Anti-inflammatory |
| ^c^CLOMIPRAMINE HYDROCHLORIDE | 3R-Tau(46)&4R-Tau(46) | 2300061 | C19H24Cl2N2 | Antidepressant |
| ^c^BENAZEPRIL HYDROCHLORIDE | 3R-Tau(39)&4R-Tau(58) | 1505200 | C24H29ClN2O5 | ACE inhibitor, antihypertensive |
| ^c^DILTIAZEM HYDROCHLORIDE | 3R-Tau(56)&4R-Tau(59) | 2300214 | C22H27ClN2O4S | Ca channel blocker, coronary vasodilator |
| SERTRALINE HYDROCHLORIDE | 3R-Tau(60)&4R-Tau(57) | 1505262 | C17H18Cl3N | antidepressant, 5HT uptake inhibitor |
| RITANSERIN | 3R-Tau(24)&4R-Tau(29) | 1503421 | C27H25F2N3OS | antiserotonin |
| CARVEDILOL | 3R-Tau(69)&4R-Tau(62) | 1504257 | C24H26N2O4 | beta-adrenergic blocker |
| LOPERAMIDE HYDROCHLORIDE | 4R-Tau(67) | 2300241 | C29H34Cl2N2O2 | Ca channel blocker |
| CANDESARTAN CILEXTIL | 3R-Tau(46)&4R-Tau(66) | 1504261 | C33H34N6O6 | angiotensin 1 receptor antagonist |
| NISOLDIPINE | 3R-Tau(52) | 1505390 | C20H24N2O6 | vasodilator (coronary) |
| BENZYDAMINE HYDROCHLORIDE | 3R-Tau(58)&4R-Tau(63) | 1505975 | C19H24ClN3O | analgesic, antipyretic, antiinflammatory |
| RIBOFLAVIN | 3R-Tau(4)&4R-Tau(5) | 1505347 | C17H20N4O6 | Vitamin B2 |
| ODORATONE | 3R-Tau(45)&4R-Tau(47) | 100093 | C30H48O4 | insecticide |
| ^a^ACETYL ISOGAMBOGIC ACID | 4R-Tau(39) | 300549 | C40H46O9 | UPR and apoptosis activator |
| DEOXYSAPPANONE B 7,4'-DIMETHYL ETHER | 3R-Tau(55)&4R-Tau(48) | 200484 | C18H18O5 | Flavon, Microtubule inhibitor |
| OBTUSAQUINONE | 3R-Tau(65) | 200090 | C16H14O3 | anti-cancer drug, cysteine reactive |
| ^b^PHENETHYL CAFFEATE (CAPE) | 3R-Tau(63)&4R-Tau(41) | 1502209 | C17H16O4 | antineoplastic, antiinflammatory, immunomodulator, NFkB blocker |
| LONCHOCARPIC ACID | 3R-Tau(6)&4R-Tau(4) | 300228 | C26H26O6 | Flavon |
| **^a,b^**beta-ESCIN | 3R-Tau(46)&4R-Tau(42) | 1504030 | C55H86O24 | membrane permeabilizer |
| ^b^ASIATIC ACID | 3R-Tau(61)&4R-Tau(55) | 1505175 | C30H48O5 | wound healing, experimental carcinogen |
| ^b^RUBESCENSIN A | 3R-Tau(52)&4R-Tau(38) | 1505177 | C20H28O6 | antibacterial, antineoplastic, insect growth inhibitor |
| METERGOLINE | 3R-Tau(49)&4R-Tau(35) | 300565 | C25H29N3O2 | analgesic, antipyretic, 5-HTR ligand |
| DEMETHYLNOBILETIN | 3R-Tau(64)&4R-Tau(59) | 1505030 | C20H20O8 | Flavon |
| CAPSANTHIN | 3R-Tau(70) | 1505276 | C40H56O3 | antineoplastic |
| ^a^NONOXYNOL-9 | 3R-Tau(45)&4R-Tau(52) | 1505292 | C33H60O10 | spermatocide, contraceptive |
| SINENSETIN | 4R-Tau(67) | 1505381 | C20H20O7 | Flavon |
| HEXAMETHYLQUERCETAGETIN | 3R-Tau(55)&4R-Tau(59) | 1505383 | C21H22O8 | Flavon |
| **^a^**TOMATINE | 3R-Tau(50)&4R-Tau(52) | 1504079 | C47H79NO21 | antifungal, antibacterial, antiinflammatory agent |
| DESACETYLCOLFORSIN | 3R-Tau(53)&4R-Tau(56) | 1503805 | C20H32O6 | Adenylate cyclase activator |
| GAMBOGIC ACID AMIDE | 3R-Tau(52)&4R-Tau(59) | 201522 | C38H45NO7 | TrkA receptor agonist |
| LANOSTEROL | 3R-Tau(64)&4R-Tau(67) | 240470 | C30H50O | Cholesterol precursor |
| **^b^**GOSSYPOL | 3R-Tau(70) | 1504019 | C30H30O8 | antispermatogenic, antineoplastic, anti-HIV |
| **^a^**HEXETIDINE | 3R-Tau(47)&4R-Tau(35) | 1500633 | C21H45N3 | antifungal |
| CYCLOBENZAPRINE HYDROCHLORIDE | 3R-Tau(67)&4R-Tau(58) | 1503207 | C20H22ClN | muscle relaxant (skeletal), 5-HT2 receptor antagonist |
| PERHEXILINE MALEATE | 3R-Tau(31)&4R-Tau(25) | 1503227 | C23H39NO4 | coronary vasodilator |
| **^b^**3-METHOXYCATECHOL | 3R-Tau(70) | 1600919 | C7H8O3 | vasodilator |
| 18-AMINOABIETA-8,11,13-TRIENE SULFATE | 3R-Tau (62) &4R-Tau(60) | 1504234 | C20H33NO4S | Leelamine, anti-cancer activity |
| VT11 | 3R-Tau (49) &4R-Tau (69) | Synthetic [Tell et al., 2012] | C21H17NO4 | GSK3 inhibitor |
| NS185 | 3R-Tau (41) &4R-Tau (54) | Synthetic (from the group of Prof.Andreas Hilgeroth) | C23H20N2O2 | JNK inhibitor |
| B6/55 | 3R-Tau (47) &4R-Tau (17) | Synthetic [Briel et al., 2009] | C10H8F3NO2S | Potential GluR5/6 antagonist |
| Stimulation |  |  |  |  |
| PROCAINE HYDROCHLORIDE | 4R-Tau (203) | 1500504 | C13H21ClN2O2 | anesthetic (local) |
| UREA | 4R-Tau (215) | 1500604 | CH4N2O | diuretic |
| ARTEMISIN | 3R-Tau (149)&4R-Tau(153) | 1500659 | C16H20O4 | anthelmintic |
| ACETYLGLUCOSAMINE | 3R-Tau(131)&4R-Tau(149)) | 1500715 | C8H15NO6 | antiarthritic |
| Note: a) Surfactant-like properties | | | | |
| b) Catechol structure element | | | | |
| c) Benzoazepin structure element | | | | |

**Suppl. table 1. Effects of selected pharmacologically active compounds from the Spectrum Collection and rationally designed inhibitors on tau self-interaction.** Tau self-interaction of 1N3R and 1N4R constructs was measured in Huh-7 cells expressing NanoBiT tau biosensors (pFN33 and pFC36 constructs) following treatment with the indicated compounds. Data are normalized to untreated control cells. Compounds reducing tau self-interaction to ≤70% (after/before) were classified as inhibitors, whereas compounds increasing interaction to ≥120% were classified as stimulators.

**References**

1.	Friedhoff P, Bergen M von, Mandelkow EM et al. (1998) A nucleated assembly mechanism of Alzheimer paired helical filaments. Proc Natl Acad Sci U S A 95:15712–15717. https://doi.org/10.1073/pnas.95.26.15712

2.	Saijo E, Metrick MA, Koga S et al. (2020) 4-Repeat tau seeds and templating subtypes as brain and CSF biomarkers of frontotemporal lobar degeneration. Acta Neuropathol 139:63–77. https://doi.org/10.1007/s00401-019-02080-2
